# Eating Time Modulations of Physiology and Health: Life Lessons from Human and Ruminant Models

**Published:** 2012

**Authors:** Akbar Nikkhah

**Affiliations:** *Department of Animal Sciences, Faculty of Agricultural Sciences, University of Zanjan, Zanjan, Iran *

**Keywords:** Eating time, Health, Human, Physiology, Ruminant

## Abstract

Tissue nutrient supply may be synchronized with endogenous physiological rhythms to optimize animal and human health. Glucose tolerance and insulin sensitivity have endogenous rhythms that are not essentially dependent on food type and eating. Human glucose tolerance declines as day comes into night. Based on such evolutionary findings, large evening meals must be avoided to reduce risks of visceral adiposity, diabetes, hypertension and related cardiovascular complexities. Ruminants as extremely important food-producing livestock have evolved to ruminate mostly overnight when little grazing occurs, and when rumen reaches a larger volume and fermentation capacity. As such, eating time (e.g., evening vs. morning) will alter postprandial and diurnal patterns of food intake, rumen and peripheral metabolites production and supply, and milk and meat production efficiency. Most recent discoveries suggest that eating time modulates postprandial intake and metabolism patterns in non-grazing lactating cows. Eating rate and absolute intake can increase by evening vs. morning feeding in dairy cows. Evening feeding increased postprandial rumen volatile fatty acids (VFA) peak, and surges of blood insulin, lactate and beta-hydroxybutyrate, and induced a peripartal decline in blood glucose. As a result, milk fat and energy production were increased. While being unfavorable to human health, evening and night feeding have proved beneficial to ruminants. These findings establish a differential chronological basis for food intake and nutrient metabolism in man and food-producing animals. Eating time is a major external cue and a feasible life strategy that affects production and health physiology.

## Introduction

**Figure 1:**
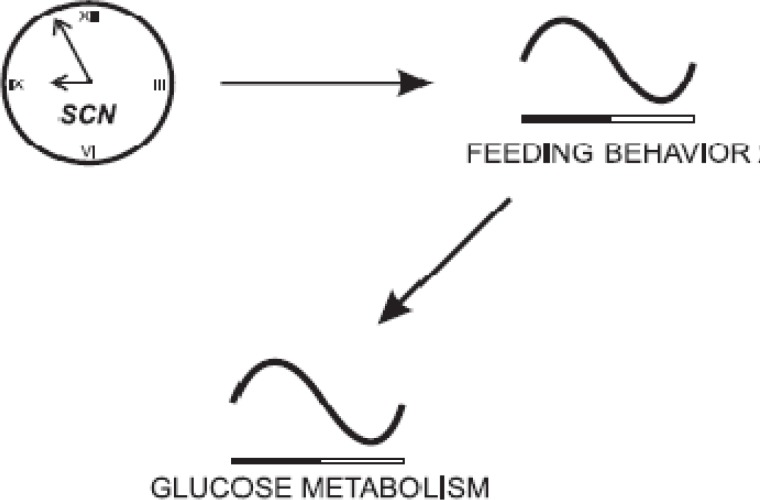
The suprachiasmatic nuclei (SCN; biological clock) driven rhythms in food intake and glucose metabolism. Black and white lines represent night and day times, respectively (15).

Tissue nutrient supply may be synchronized with endogenous physiological rhythms to maximize nutrient efficiency and optimize metabolic heath. Such biorhythms are closely linked to eating patterns (1-3). Postprandial eating patterns determine daily eating behavior in ruminants and humans (4, 5). Circadian rhythms of cell metabolism are shown in endogenous and exogenous rhythms of blood metabolites and hormones (6, 7). Endogenous rhythms are controlled by the hypothalamic suprachiasmatic nuclei (Figure 1), and not only by the environmental factors such as photoperiod and feeding timing (8). Glucose tolerance and insulin action are regulated endogenously (9, 10). In contrast, exogenous rhythms are controlled mostly or entirely by external cues. Blood urea in goats, for instance, is largely responsive to feeding and digestion and is, thus, regulated exogenously (7). Feeding time is a farm strategy that can alter post-feeding rhythms of ingestion, nutrient assimilation, and peripheral metabolite supplies (11-13). Such reflections will indicate optimum times of the 24 hr period when nutrients can be processed more efficiently for productivity and health. A primary objective of this review article is to integrate most recent discoveries on eating time's modulation of human and ruminant physiology. Another objective is to lead such integrations into development of feasible life strategies that can improve metabolism and health of humans and high-producing ruminants that are supposed to supply humans with adequately safe and secure food resources. Such conclusive perspectives are essential for the timely public education of optimum nutritional programs, given the exposure to a variety of environmental stressors in the new era. 

***Human physiology models highlights***

**Figure 2:**
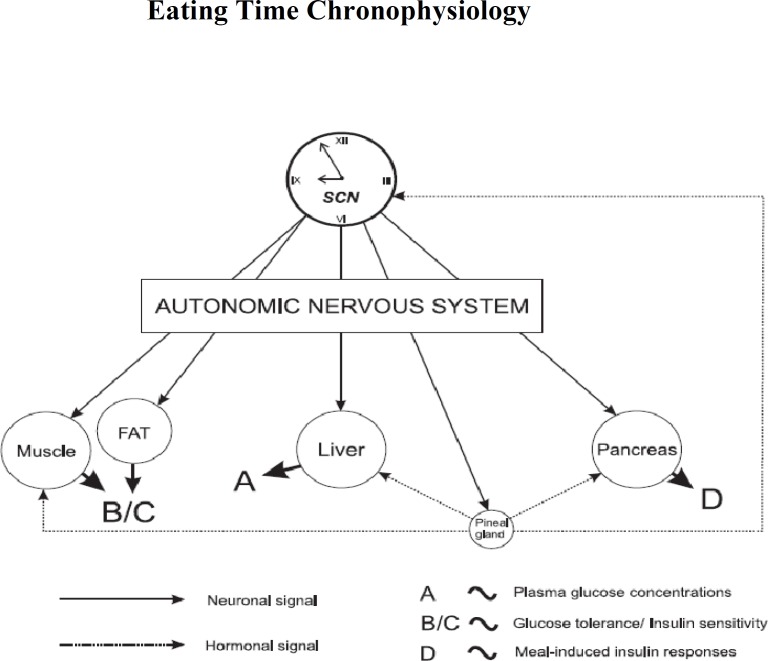
A model for the suprachiasmatic nuclei (SCN)-mediated regulation of glucose metabolism in human (9, 10)

Blood glucose in humans and rats has endogenous rhythmicity (9, 10). This means that insulin sensitivity and glucose tolerance vary depending on time of the 24 hr period, regardless of when eating occurs (10, 14). Glucose tolerance is the relative amount of glucose taken up by peripheral tissues. Increased glucose tolerance, which means a higher glucose uptake, results from 1) a higher amount of insulin secreted from the pancreatic beta-cells, 2) increased glucose transporters, and 3) a higher availability (specificity) and sensitivity (affinity) of insulin receptors. Thus, insulin insensitivity is the increasing insulin quantity required to maintain euglycemia. Insulin insensitivity is a result of reduced availability or sensitivity of insulin receptors, which reduces peripheral glucose uptake and impairs the inhibition of hepatic glucose synthesis (9). From an evolutionary viewpoint, humans cannot metabolize glucose as effectively in the evening as they can in the morning. These are at least in part because glucose is demanded most during more active times of the day. Hence, glucose tolerance and insulin efficiency decline as day comes into night (15) (Figure 2). Such evolutionary insights are integrated into a recommendation to avoid large evening meals to reduce risks of visceral adiposity, diabetes mellitus, and cardiovascular disorders. Shift workers should thus be under special nutritional regimens to not overeat overnight to minimize such risks. 

**Figure 3:**
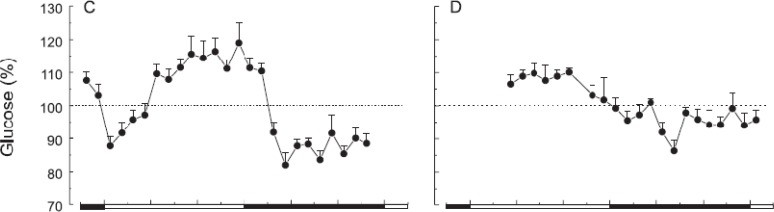
Basal peripheral blood glucose concentrations (as % of the 24 hr mean±SEM) across the light-dark cycle in intact rats (left graph; n=8) and suprachiasmatic nuclei (SCN)-lesioned rats (right graph; n=7) under fasting conditions. The black line areas are night times (15)

Circadian rhythmicity in human glucose metabolism gives rise to blood glucose rhythms that are not essentially food-driven (Figures 1-3). This means that even during fasting, blood glucose exhibits distinct of the 24 hr patterns in human blood glucose notwithstanding eating regimen emphasizes the endogenous nature of blood glucose regulation (16). The suprachiasmatic nuclei regulate blood glucose independent of periprandial food intake patterns (15) (Figure 3). 

Melatonin may be involved in food intake and glucose metabolism in humans and rats (9, 17, 18). For instance, exogenous melatonin dosed via drinking water has increased postprandial insulin response (10, 15). Melatonin secretion is basically induced by darkness. In humans, reduced nocturnal glucose tolerance is concurrent with increased melatonin secretion (16). Reduced glucose tolerance is due to reduced insulin reaction, and reduced peripheral glucose uptake. These reflect reductions in glucose and insulin requirements (14, 19), which are biologically meaningful since glucose should be least required at times of inactivity or night. The increased blood glucose could, thus, indicate a decline in peripheral glucose uptake and insulin turnover (17).

***Insulin and food ingestion: inter-species intuitions***

Insulin as the main storage hormone stimulates glucose entry into peripheral adipocytes and muscle cells (20, 21). Less glucose enters portal vein in ruminants vs. humans (22). As a result, insulin may not have as significant effects on hepatic glucose uptake and metabolism in ruminants as it does in humans. Nervous system, gut peptides, pancreatic secretions, and nutrient absorption all induce insulin release (23, 24). Nervous-wise, insulin is released by the action of sympathetic and parasympathetic neurons. Food vision, odor, and flavor can also induce insulin secretion by activating parasympathetic neurons in humans. Earlier fundamental research (25-27) established that neural impulses and gastrointestinal hormones are involved in ruminant postprandial insulin responses. Secretin and pancreozymin stimulated insulin release in sheep (27). The blood insulin rise precedes that of glucose, suggesting that glucose is not necessarily a major cause of the initial postprandial rise in insulin release (28, 29). According to the most recent discoveries, such effects depend on eating time. Cows fed once daily at 2100 hr exhibited a pre-feeding decline in blood glucose that progressed until 2 hr post-feeding before reaching the baseline at 4 hr post-feeding (12, 13). In 0900 hr-fed cows, however, blood glucose remained constant without such a distinct peri-feeding rhythm (13). As such, postprandial insulin surges were higher for evening vs. morning feeding (30, 31). Thus, insulin action and glucose uptake relations to food provision and eating depend on when eating occurs. 

Food presentation and milking both induce eating in individually-fed and group-housed dairy cows (32, 33). Food provisions effects on eating activity may well persist even with multiple daily feeding (4). The literature suggests that dairy cows eat when fresh food is offered and that the amount eaten after food delivery depends on time of day. Anticipation of food presentation time may elongate eating time in cows (34). Increased eating rate shortly after evening food provision (5, 31) suggests that cows may anticipate evening feeding better than morning feeding. Plasma insulin was higher and glucose was lower at 2 hr post-feeding in evening vs. morning fed cows (30). Higher insulin could weaken glucagon, thus reducing gluconeogenesis (20). The intravenous glucagon has reduced food intake in sheep (35). It is, therefore, likely that higher blood insulin and lower glucose at 2 hr post-feeding in evening fed cows may delay the glucagon-driven satiety and increase postprandial eating rate (13).

In goats fed *ad libitum* for only a 3 hr period daily, a post-feeding rise in blood insulin occurred (36). This is probably induced by volatile fatty acids (VFA) affecting the pancreatic beta-cells. On the other hand, de Jong (37) observed a post-meal rise in blood insulin of goats without essentially a blood VFA peak. Thus, nervous signals rather than VFA alone appear directly or via gut hormones to elicit a post-meal insulin response. In lactating cows fed once daily at 0900 hr, blood insulin exhibited distinct diurnal rhythms, with a peak at 1745 hr and a nadir during 2300-0700 hr (38). A similar blood insulin peak occurred at 1830 hr (39) and at 1800 hr (40). Thus, diurnal rhythms of peripheral blood insulin are closely related to eating patterns. Diurnal patterns of peripheral blood insulin are more closely related to food content of non-structural carbohydrates, such as sugars and starch. 

***Ruminant physiology: evolutionary insights into eating time***

Evening instead of morning food provision improved beef cattle performance (41, 42). Lactating cows are extraordinary mammals with exceptionally high intake and production levels above maintenance (43). Chronobiological mediations of rumen and cow metabolism may hence largely affect eating behavior and diurnal rhythms of rumen, portal, and peripheral blood metabolites. These will in turn affect milk biosynthesis and tissue energy turnover (5, 31). Blood glucose exhibited significant periprandial responses to food delivery at 2100 hr but not at 0900 hr (5, 30). 

**Figure 4:**
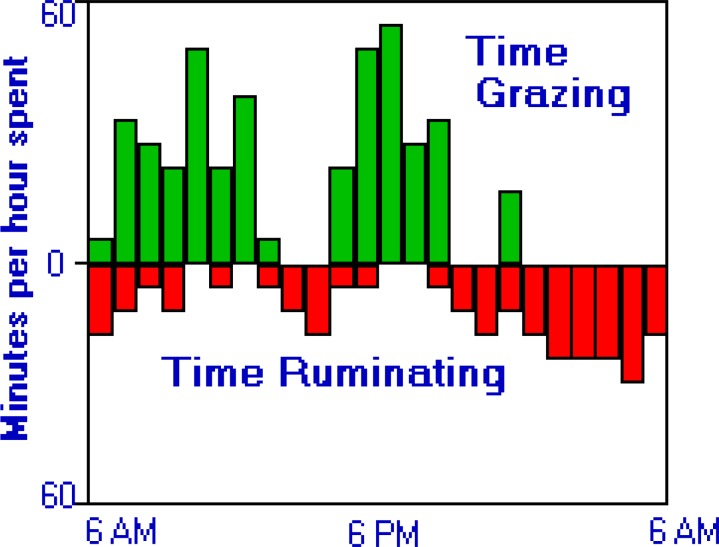
Diurnal patterns of rumination and eating in steers grazing alfalfa pastures (1, 2, 60)

**Figure 5:**
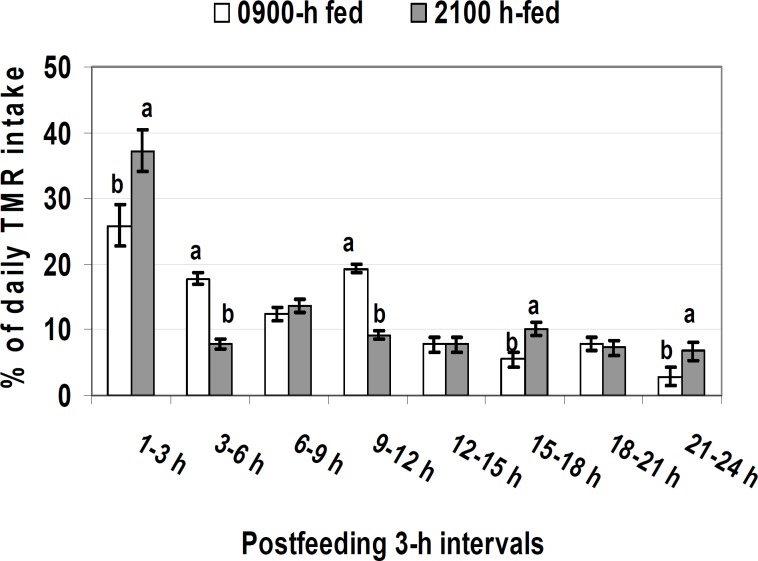
Postprandial food intake patterns in cows fed at either 0900 hr or 2100 hr. Within each 3 hr, bars with different superscripts differ at *P* < 0.05 (1, 2, 5).

**Figure 6:**
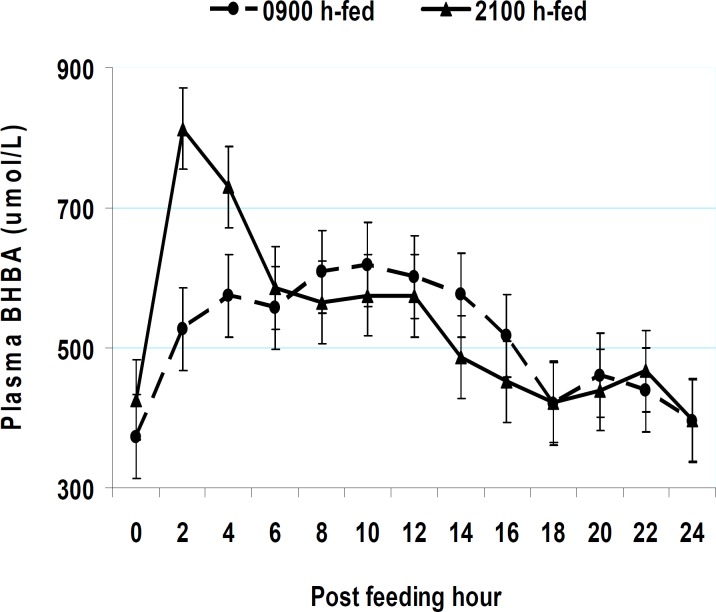
Postprandial patterns of plasma lactate and beta-hydroxybutyric acid (BHBA) in cows fed at either 0900 hr or 2100 hr. Within each sampling time, * = *P* < 0.05 (1-3, 59)

**Figure 7:**
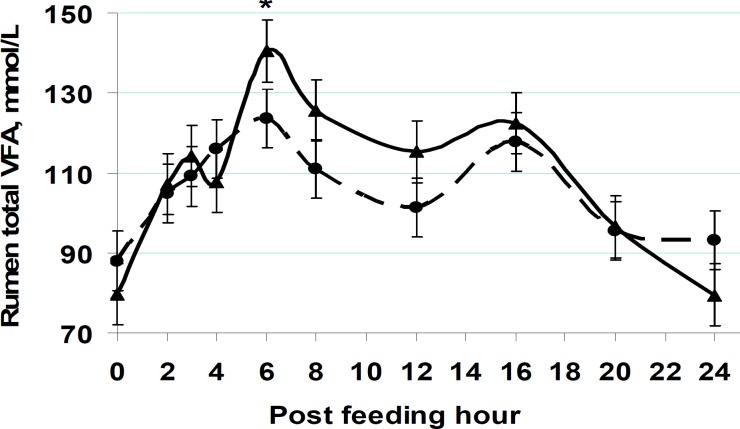
Postprandial rhythms of rumen pH and total volatile fatty acids (VFA) concentrations in cows fed at either 0900 hr or 2100 hr. Within each sampling hour, * = *P* < 0.05 (2).

Chronobiological mediations of rumen and intermediary metabolism are expected to affect cow physiology and milk production. Ruminants have evolved to ruminate mostly overnight when little grazing occur and when the rumen has been found to have a greater volume than day-time (2, 44) (Figure 4). This evolution is consistent with the more nutritious evening vs. morning pastures due to day-time photosynthesis in plant leaves (45, 46). As such, feeding during night hours, when ruminants have evolved to actively ruminate, altered postprandial eating patterns, VFA and glucose metabolism, and thereby peripheral nutrient supply (Figures 5-7).

In humans and rats, regulation of glucose metabolism and insulin sensitivity depend heavily on time of day (9, 10, 14). Humans are unable to metabolize glucose effectively during evening hours because insulin action diminishes as day progresses and evening begins (15). Accordingly, large evening meals must be avoided should reducing risks of diabetes mellitus and related cardiovascular complexities be aimed. 

It was only recently that feeding time effects on postprandial patterns of intake, rumen fermentation, and key blood metabolites in lactating cows under thermoneutral conditions were revealed (Figures 5-7). Dairy cows fed at 2100 hr and 0900 hr ingested respectively 7.6 and 5.3 kg food within 3 hr of feeding a diet with 31.2% neutral detergent fiber (NDF) and 37.8% non-fiber carbohydrate (NFC) (31). On a less starchy diet, the respective intakes were 11.2 and 9.0 kg (5). Hence, regardless of diet composition, evening eating increased ingestion rate shortly post-feeding. Lactating cows could eat as much as 50% of their daily intake only within 3 hr of feeding. Evidently, some individual cows were able to eat as much as 70% of their daily intake within only 3 hr of feeding. The greater diet fermentability may reduce intake via rumen VFA and ammonia accumulation (47). As such, the food amount ingested within 3 hr of feeding was greater with more fibrous diets. Rumen volume was greater in cows fed at 2100 hr vs. 0900 hr (1, 2, 5). The cow’s tendency to ingest more food when fed at 2100 hr vs. 0900 hr suggests that the magnitude of gut-fill effect on intake regulation differs between morning and evening. These discoveries are supported by implications that food intake is regulated by a multitude of diet, animal, and environmental factors (48-50).

The greater appetite following night eating might also partly be due to quieter farm environment. Moreover, melatonin regulates glucose metabolism (10, 17, 18) and thereby may contribute to food intake regulation. Usually, melatonin is secreted in the absence of light. Evening eating will, thus, coincide with elevated melatonin secretion. In humans, reduced nocturnal glucose tolerance is associated with increased melatonin secretion (16). Assuming a relationship between melatonin and glucose metabolism in ruminants also, diurnal food intake patterns will depend on when food is presented. 

Peripheral metabolites such as glucose and VFA depress food intake mainly via cell entries and not necessarily by staying in the blood (51). Hence, factors reducing peripheral metabolite uptake can in turn attenuate such a metabolite-driven satiety. As such, the expected rise in evening blood melatonin might reduce peripheral metabolite uptake in insulin-sensitive tissues in favor of milk biosynthesis. The higher nocturnal melatonin might then weaken the food -driven satiety in evening fed cows. This cascade is consistent with the increased eating rate shortly post-feeding in evening fed cows (2, 5, 31). Such altered intake regulation might allow a possibility for night-time glucose intolerance in lactating cows (30). Milk energy output was increased by evening instead of morning eating. This suggests that at times of greater food intake and peripheral metabolite supply, milk precursors (e.g., lactate, glucose, and beta-hydroxybutyric acid (BHBA)) were taken up more effectively by the mammary tissue. The higher postprandial rumen VFA and blood insulin surges by evening vs. morning eating concur with increased milk energy output in the evening fed cows (Figure 7). 

In another study, lactating cows were fed 67% of their diet at 0800 hr and 33% of it at 1800 hr (52). In addition, a protein supplement was offered at 15% of daily food intake at either 0830 hr or 0030 hr. Cows ate more of the protein meal when it was offered at 0030 hr vs. 0830 hr. Consequently, total food intake was increased (16.92 vs. 15.94 kg/d), which led to increased rumen dry matter (DM) and protein digestibility (52). These suggest that the midnight instead of morning protein meal eating stabilized rumen fermentation. In a heat stress study, lactating cows were fed 4 times daily different food proportions either during day as 30% at 0615 hr, 20% at 1000 hr, 25% at 1530 hr, and 25% at 1900 hr, or during evening as 20% at 0615 hr, 30% at 1530 hr, 25% at 1900 hr, and 25% at 2100 hr (53). The evening-fed group had no access to food for 5.5 hr during day. The limited food access during day and shifting eating time to evening time reduced daily food intake and energy expenditure. In freezing cold weather, feeding at 2000 hr vs. 0900 hr improved beef steers growth rate without affecting food intake (42). In another beef study (41), heifers fed at either 0900 or 2000 hr in a freezing winter were similar in food intake, while evening fed cows had higher food efficiency. 

***Eating time effects on ruminant production***

Midnight instead of morning delivery of a protein meal improved milk fat production, most likely by increased ruminal nutrient digestion (52). No such an effect was observed in a similar study (54) where lactating cows were fed a protein supplement at about 12% of daily intake either at 0830 or 0030 hr. In a 118-d lactation trial, Aharoni *et al* (53) fed heat-stressed lactating cows 4 times during either day (30% at 0615 hr, 20% at 1000 hr, 25% at 1530 hr, and 25% at 1900 hr) or evening (20% at 0615 hr, 30% at 1530 hr, 25% at 1900 hr, and 25% at 2100 hr). Shifting food delivery times from day into evening improved lactation persistency and energy efficiency (53). Others (41, 42) found that feeding at 2000 hr vs. 0900 hr improved growth in beef heifers and steers under freezing winters. Most recently, provision of higher and lower concentrate diets once daily to lactating cows at 2100 hr vs. 0900 hr increased milk fat and energy yield (31). The result was substantiated in a following study (2, 11). Energy corrected milk yield was increased by 2.1 kg/d in primiparous cows and by 1.3 kg/d in multiparous cows by evening vs. morning eating. The greater milk fat was likely due to increased rumen VFA levels and higher postprandial peaks of peripheral blood lactate and beta-hydroxybutyrate (Figures 6, 7). These were all linked to increased eating rate shortly post-feeding in evening vs. morning fed cows (Figure 5). In beef cattle, total daily intake and food efficiency have increased by evening feeding (55, 56).

***The science of evolution to optimize rumen and ruminant health***

Based on an evolutionary concept, rumination occurs mostly overnight (Figure 4). This implies a greater rumen digestion capacity overnight than during day. Such a greater night-time rumen volume and fermentation were recently revealed in grazing (57) and tie-stall housed (1, 2) lactating cows. Should the increased rumen fermentation capacity be concurrent with increased chewing activity and rumination, optimum rumen pH and microbial metabolism may be more feasible to secure with evening vs. morning feeding. Increased milk fat by night eating suggests stabilized rumen conditions (31). By increasing rumen absorption capacity, evening eating may not durably lower rumen pH to the range in which microbial rupture, endotoxins release, and proinflammatory responses occur (e.g.,< 5.2-5.5) (58).

Endocrinologically, insulin stimulates peripheral nutrient uptake. Insulin, however, does not have a major impact on propionate-driven hepatic gluconeogenesis and mammary nutrient uptake (20, 21). Thus, increased peripheral blood concentrations of various substrates by evening vs. morning eating can increase mammary and non-mammary nutrient flow and uptake. As a result, milk secretion and peripheral nutrient retention may simultaneously improve as shown previously (11, 31).

***Conclusions and implications***

Metabolic chronophysiology is of great importance with regards to eating time effects on the health of human and food-producing livestock. With glucose tolerance declining as day comes into night, large evening meals must be avoided to reduce risks of visceral adiposity, diabetes, and consequent cardiovascular complexities. Such insights have important implications for shift workers and those under special nutritional regimens. Evening vs. morning feeding of lactating cows under thermoneutral conditions has increased eating rate, thereby increasing total daily intake in given groups of cows. Evening feeding has also increased rumen volume and postprandial rumen and peripheral metabolite surges. As a result, evening feeding has improved nutrient digestibility and milk production. Ruminant evolution to graze mainly around sunrise and sunset and to ruminate mostly overnight offers perspectives to manipulate rumen volume, nutrient partitioning, and ruminant health. Manipulating eating time can help to improve production efficiency of food-producing animals whilst optimizing human health.
